# O11.7. DISCHARGE PLANNING PRACTICES AND FAMILY INVOLVEMENT IN TRANSITIONS TO OUTPATIENT CARE FOLLOWING DISCHARGE FROM HOSPITAL PSYCHIATRIC UNITS

**DOI:** 10.1093/schbul/sby015.267

**Published:** 2018-04-01

**Authors:** Morgan Haselden, Thomas Smith

**Affiliations:** Columbia University & New York State Psychiatric Institute

## Abstract

**Background:**

Individuals with mood and psychotic disorders treated in hospital psychiatric units have high rates of discontinuing treatment following discharge, a time that poses substantial risks of serious and even life threatening adverse outcomes. Hospital provider care transition practices believed to improve transitions include communication with outpatient providers, scheduling timely appointments for outpatient follow-up care, forwarding case summaries to aftercare providers, and involving family or support persons in discharge planning. While these are standards of care, little is known about how often they are adequately delivered and their impact on post-discharge aftercare adherence.

**Methods:**

As part of a larger project looking at over 30,000 hospital admissions of Medicaid patients with serious mental illness, this study examined hospital medical records for 217 admissions at two urban US hospitals. Trained raters reviewed records for evidence of inpatient providers completing discharge planning practices. Medicaid data were used to measure demographics and attendance of seven- and 30-day outpatient appointments.

**Results:**

The sample of 217 admissions was 51% male and 82% were adults, with discharge diagnoses including schizophrenia and related disorders (45%), bipolar disorders (28%) and depressive disorders (17%). The average length of stay was 14 ± 13 days with a median of nine days. The medical records showed evidence of inpatient providers communicating with outpatient providers 64% (n=139) of the time. There was evidence of an outpatient appointment scheduled within seven days of discharge for 81% (n=176) of the sample. A case summary was made available to the aftercare provider within one day of discharge for 66% (n=144) of the sample. Records showed that the inpatient team communicated with family members or support persons about the patient’s post-discharge treatment plan for 53% (n=114) of the sample, and 36% (n=79) attended a family meeting or therapy session. Rates of attending an aftercare behavioral health appointment were 55% (n=120) at seven days post-discharge and 80% (n=174) for 30 days.

**Discussion:**

This study found varying rates of providers completing care transition practices. Only half of the sample had attended an aftercare appointment in the seven days post discharge, however the majority had attended an appointment by 30 days. Planned analyses will present demographic and clinical differences among those who received discharge planning activities and had family involvement. We will examine predictors of attending follow-up care and report the effectiveness of discharge planning practices. Findings will help inform strategies to improve care-coordination and discharge planning for individuals with serious mental illnesses treated in psychiatric hospitals.

